# Optimal patient classification via statistical decomposition of a 3D left-ventricular atlas

**DOI:** 10.1186/1532-429X-15-S1-P89

**Published:** 2013-01-30

**Authors:** Pau Medrano-Gracia, Brett R Cowan, David A Bluemke, J Paul Finn, Daniel C Lee, Joao A Lima, Avan Suinesiaputra, Alistair A Young

**Affiliations:** 1 Auckland Bioengineering Institute, The University of Auckland, Auckland, New Zealand; 2 NIH Clinical Center, Bethesda, MD, USA; 3 Feinberg Cardiovascular Research Institute, Northwestern University, Chicago, IL, USA; 4 Donald W. Reynolds Research Institute, Johns Hopkins University, MD, USA; 5 Diagnostic Cardiovascular Imaging, University California Los Angeles, Los Angeles, CA, USA

## Background

**Figure 1:**
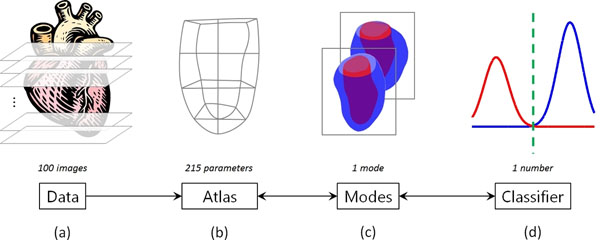
Classification framework developed for this work: (a) cardiac MRI data were segmented and fitted to LV models; (b) models were aligned to build an atlas; (c) different modes of variation were derived and (d) optimal classifiers were analysed in terms of shape and accuracy.

Statistical quantification and classification of heart disease, using clinical indices such as ejection fraction (EF) or left-ventricular mass (LVM), are used routinely in clinical practice for both diagnosis and prognosis. However, 3D CMR of the left ventricle (LV) provides a wealth of shape features which can maximise the classification power and accuracy of such indices. To exploit these, we propose a framework whereby shape parameters of a 3D LV finite element atlas are used to optimally classify patients according to linear statistical decompositions (Fig. 1).

## Methods

Finite-element shape models of the LV were customised to 600 cardiac MRI volumes with previously standardised and validated software (CIM v. 6.0, Auckland, NZ). The dataset comprised 300 community-based participants without a history of cardiovascular disease, aged 45-84 from 4 ethnic groups from the *Multi-Ethnic Study of Atherosclerosis* (MESA) cohort (1) and 300 patients with myocardial infarction from the *Defibrillators To Reduce Risk By Magnetic Resonance Imaging Evaluation* (DETERMINE) cohort (2) made available through the Cardiac Atlas Project (3). Bias due to scan protocol differences between cohorts was corrected (4). Shape classifiers were constructed to optimally detect which cohort each case belonged. A comparison between principal component analysis (PCA) and information preserving component analysis (IPCA) (5) was performed, using shapes at end-diastole (ED), end-systole (ES) and the difference in shape between ED and ES (ED-ES) which included information on regional wall motion. Traditional clinical classifiers of EF, end-diastolic/end-systolic volume (EDV/ESV) and LVM were also included for comparison. Ten-fold cross-validation experiments were performed in which 90% of the cases were used for training and 10% for validation, repeated 10 times with different training/validation cases.

## Results

Classification results showed that this framework was able to determine clinically relevant modes and that IPCA achieved the lowest error rates using the ED-ES shape difference, with a single classifier number. This classifier can also be used to quantify severity of disease (degree of match with each group). Ten-fold cross-validation experiments corroborated the robustness of this approach which averaged 100% specificity and 99% sensitivity for IPCA versus 83% and 93% respectively when compared to ejection fraction (Table 1). Further, by back-projecting the optimal classifier onto the atlas, we were able to quantify and visualise which regions of the LV had the most weight in the decision. For the ED-ES classifier, this was a combination of volume and wall thickness change.

**Table 1:** Specificity and sensitivity are shown in brackets (in that order) for the cross-validation experiments.

	ED	ES	ED−ES
PCA	(53.8,54.4)	(77.5,75.6)	(89.4,87.9)
IPCA	(96.6,91.2)	(96.2,98.0)	(100,99.0)

EF			(82.7,93.2)
EDV	(81.6,92.4)		
ESV		(79.2,98.3)	
LVM	(67.1,70.1)		

Columns indicate the type of information used by the classifier; rows indicate which classifier is used. Top two rows are different statistical decomposition techniques of shape parameters (preserving only 1 dimension) and bottom four rows are traditional clinical heart-failure indicators. In the case of ED&#8722ES, PCA and IPCA include information on regional wall motion.

## Conclusions

This work shows the potential of shape based classification in the automated identification and quantification of heart disease.

## Funding

This work was supported by award no. R01HL087773 from the NHLBI. The content is solely the responsibility of the authors. The NIH (5R01HL091069) and St. Jude Medical provided grant support for the DETERMINE trial. MESA was supported by contracts N01-HC-95159 through N01-HC-95169 from the NHLBI and by grants UL1-RR-024156 and UL1-RR-025005 from NCRR.
